# Supplementary material for: MetaRibo-Seq measures translation in microbiomes
Source: Nat Commun. 2020 Jun 29;11:3268. doi: 10.1038/s41467-020-17081-z (PMC7324362; doi:10.1038/s41467-020-17081-z)
Supplement: Supplementary file 10 — Supplementary Data 7 [file 41467_2020_17081_MOESM10_ESM.zip › File2/Confidence_High_Taxonomy/296550_out.krona.html]

Javascript must be enabled to view this page.

members
magnitude
magnitudeUnassigned
count
unassigned
taxon
rank

296550\_out

3

2
superkingdom
3

1239
3
phylum

3
class
186801

3
order
186802

species

SRS049446\_contig\_number\_9031
1
1950889

541000
family
2

1
species

SRS893256\_contig\_number\_1579
1627893

216851
1
genus

1946508

SRS020328\_contig\_number\_32448
species
1
